# Improving the pilot selection process by using eye-tracking tools

**DOI:** 10.16910/jemr.12.3.4

**Published:** 2020-02-18

**Authors:** Slaviša Vlačić, Aleksandar Knežević, Sanja Rođenkov, Saptarshi Mandal, Panos A. Vitsas

**Affiliations:** Military Academy, University of Defense, Belgrade, Serbia; Aeromedical Institute, Belgrade, Serbia; Industrial & Systems Engineering, University of Oklahoma, USA; ITPS, London ON, Canada

**Keywords:** Eye movement, eye tracking, gaze, attention, fixation, revisit, areas of interest, flight screening

## Abstract

This paper improves the understanding of the use of eye-tracking tools in the pilot selection process. Research of eye movement and attention distribution of candidate pilots may provide the capability for visual behavior prediction in more demanding flight training phases. The research included psychological testing, flight screening of subjects and their achievements in a flight simulator in combination with an eye-tracking device. Participants were divided into three categories: high performance, average performance, and low performance and separately regarded through psychological testing results and flight screening results. An eye-tracking device tracked visual behavior of subjects through the scope and speed of visual perception. The number of fixations and revisits recorded during the simulated visual flight conditions measured the difference in visual response between subjects. Comparison of results showed a positive correlation with psychological test results. Correlation with flight screening selection was not confirmed. We used the new network-based approach with three target importance measures to overcome the shortcomings of traditional eye movement metrics. The results of the adopted network approach presented in the form of graphs and analysis of normalized importance measures showed that it was possible to extract specific saccade strategy for each participant. Discovered differences between them positively detected week ones. In this way, Eye-tracking tools can potentially improve the pilot selection process and complement other tests and assessment methods.

## 1 Introduction

The selection of military pilots is a lengthy, complex and expensive process^(1)^. The main goal of this selection process, regardless of their phase (i.e. primary, secondary or tertiary) is to filter out appropriate candidates (cadets or professional pilots) who can fly some particular fast jet airplanes, e.g. F-16, MiG-29 and/or helicopters. The skills required to become a pilot defined by medical and psychological parameters, besides perfect health, requires each candidate to possess high levels of general and specific abilities and personality pattern, e.g. making judgments under stress, confidence etc. All the above-mentioned characteristics are tested during the basic training process. The student pilots should have the ability to successfully cope with the flight syllabus and develop the required pilot aptitudes (e.g. understanding & remembering the procedures, visual orientation, regularity in procedures and operations)^(2)^.

To shorten the training cycle, improve its efficiency and save expenses, the initial training of the novice candidates takes place on propeller-driven aircraft, rather than jet combat airplanes. In addition, Wang and Chan^(3)^ noted in that the candidates’ initial training performance and the systems used to evaluate it plays critical role in determining their future flight training success. Moreover, it is to be noted that the transition between different aircraft systems (used for training process) brings in different visual interfaces and instruments inside the cockpit^(4)^. As a result, it becomes imperative to understand how these introduction of new aircraft (equipped with digital cockpit instruments) affects an existing training process, and subsequently the selection process. This also raises an important question whether a different approach is required to evaluate the candidates’ aptitude.

To provide an overview for the uninitiated, the selection process consists of three overall phases. During the first phase, the candidates must undergo a demanding medical, psychological (measuring attention capacity, logical reasoning, mental speed, perceptual reasoning, memory, and spatial ability) and psychomotor test (involving a computer-based test and measurement of eye-hand-leg coordination). The psychological tests are performed prior to the flight screening test. In detail, reasoning tests T2 and T5 tests, and Wulfften-Palthe (WP) (both paper and pencil-based tests) are used to evaluate the candidates attention and perception abilities^(5),(6),(7)^. The second phase (also called flight screening) consists of the initial flight training in a light piston-engine aircraft. In this phase, different skills such as working and intellectual skills, perceptive and emotional senses are evaluated^(2)^. Introduction of more advanced aircraft for the flight screening phase results in both increase in operational complexity and cost per flight hour. Thus, it becomes essential to enable earliest possible detection of inappropriate candidates. On the other hand, elimination of candidates in later stages can result in significant financial losses in the range of hundreds of thousands of dollars, especially if the candidate has already begun flying in fast jet trainers. As a result, we need efficient and low-cost performance evaluation methods, which are in accordance with the traditional methods, to filter our incompatible candidates in the earlier stages of the screening process. To this purpose, many previous studies have advocated the use of eye tracking tools, applied mostly in simulated flight scenarios^(8),(9),(10)^. Some of these studies analyzed the pilots’ visual scanning strategies for different flight scenarios and for various pilot categories^(11),(12),(13),(14)^.

In the third selection phase, the cadets are evaluated based on their aerobatic flying skills (e.g. vertical maneuvers, submission to g-forces) on a light one engine piston aircraft. In this phase, the high performing cadets are sent for further training in fast jet aircraft, average performers are sent for helicopter training, and the low performance cadets are eliminated from further training.

It’s evident from the above-mentioned description of the various screening phases that it’s very costly training process. Thus, it becomes imperative to filter our unfit candidates as early as possible to reduce the training cost involved. To this purpose, a cheaper alternative approach that can predict a candidate’s performance becomes very handy. Many previous studies have shown that eye tracking is a viable cheap alternative that can analyze pilot’s visual scanning strategy inside a cockpit, thus enabling their performance evaluation,^(15),(16),(17)^. We should note that a substantial part of the pilot selection tasks involves visual information gathering; thus, it is judicious to apply eye tracking to evaluate the potential candidates’ performance. However, there exists some issues in terms of the validity of the EM results in such a setting and how it can facilitate the pilot selection process, e.g. where should we place the EM studies.

Given the three above-mentioned screening phases, can it be used to pre-screen candidates even before they undergo first screening phase, or should it be used to compliment the psychological screening phase to eliminate low performing candidates from further screening, thereby reducing training cost. To answer these pertinent questions, the primary objective of the present study is to analyze the correlation between the various EM measures (obtained during simulated flight operations) and the traditional psychological test results, and in-flight evaluation of student pilots. Furthermore, the secondary objective of the study is to evaluate whether EM analysis can be used to categorize potential candidates into different performance groups (high, medium, and low) by analyzing their visual scanning strategy while they operate technically advanced aircraft. A concurrence between the EM analysis are their traditional counterparts would further validate the use of low-cost eye tracking tools in the pilot selection process for digital avionics cockpits resulting in a significant cost saving.

To this purpose, presently we focus on the comparative analysis of the visual scanning strategy of the novice candidates’ only (unlike previous studies where pilots were the focus of study, (see Table 1), who are one step behind the beginners in the initial flight training. Moreover, we analyzed the novice candidates’ EM data for five different flights tasks (e.g. take-off, climb, turn, straight-and-level flight, and landing) in three sorties of Visual Flight Rules (VFR).

Furthermore, we also explored whether EM analysis can help us further simplify the training process by reducing the amount of time required in the simulated flight scenarios. To address this, we proposed an-depth network-based EM analysis which helps to evaluate the dynamic behind deployment of visual attention by the candidates, especially during ‘take-off’ and ‘landing’ phases of the flight simulation tasks (considered most critical phases). If the candidates fail to provide adequate attention on the important regions on the display during these two critical phases, then they get very low overall scores irrespective of their performance in other flights phases. This methodology will thus help us in cutting short the training time and eventually further cost reduction. Thus, to understand the dynamics of the visual attention deployment strategy, we have used the network-based EM analysis that includes visualizing the EM data in the form of a directed weighted network and further develop advanced EM measures using network-based importance metrics, e.g. Indegree, Closeness, and Betweenness (described in detail in background section) ^(18),(19)^.

In this paper, we propose a new methodology, combining traditional psychological test (paper and pencil-based) performed in a controlled environment and eye tracking methodology (collected during simulated flight) and live training, to address the prevalent issues in pilot selection process. First, we investigated the use of the EM analysis as a useful prediction tool concerning the candidates' achievements in the psychological tests. Second, we have analyzed the possible correlation of EM data with the flight screening outputs. Third, for unraveling the dynamics of the visual scanning strategy of the candidates’ we adapted the network-based representation of the visual scanpath and also evaluated the various advanced EM measures that enables understanding the changing importance of various regions on the display, from visual scanning strategy respective, applicable in case of a dynamic tasks (flying an aircraft). This was also complimented with the bar plots of the EM measures that enables a comparative analysis of the differences in visual scanning strategy among the candidates and how it evolves over the various flights phases for a given candidate.

## 2 Background

### 2.1 Psychological selection test

We used the Aeromedical institute archive data to introduce the study of the visual behavior of nine participants in the laboratory environment, during psychological selection (Figure 4).

The psychological selection phase consists of three tests namely, T2, T5 and Wulfften-Palthe (WP)^(5),(6),(7)^. These tests are part of a multiple aptitude battery tests used for evaluating candidate’s visual perception ability and attention capacity. It has been used for the selection of the Serbian Air Force cadets for more than three decades. T2 and T5 tests evaluate a candidate’s visual perception, where T2 is used to evaluate the speed of visual reasoning, and T5 is measures speed of perceptive thinking in obtained situation.

**Figure 1:**
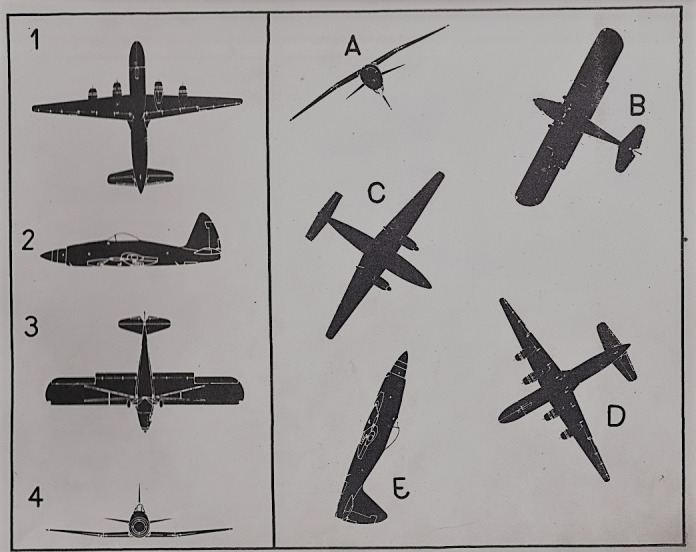
Example layout of various aircraft representation used for T2 test.

**Figure 2:**
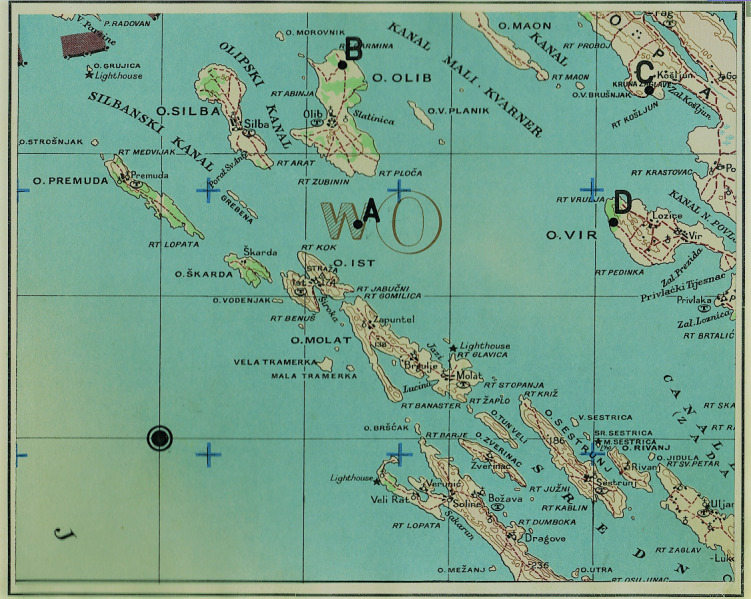
Example layout of topographic map with focal point used for T5 test.

T2 test consists of 52 tasks with increasing complexity. The subjects were asked to locate four identical figures of airplane specified in a group of five. The goal is to match identical plane by letter and number (eg.1-D, 2-E, 3-B, 4-A) within a time limit of 12 seconds/task. Figure 1 shows an example layout of the display used for the T2 test. On the other hand, T5 test consists of 48 items with increasing complexity and with a time limit of 4 minutes. The subjects meet clippings, topographic maps with one focal point and a growing number of peripheral dots. The goal is to determine, with quick and accurate visual search, which peripheral dots are closer to the focal point (e.g. A-B-D-C). Figure 2 shows the display layout used for T5 test.

**Figure 3:**
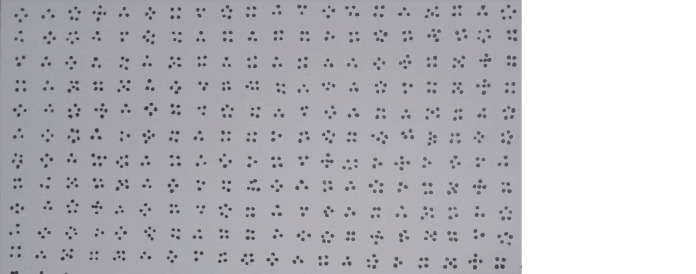
Example of the WP test (part of the sheet used for WP test).

WP (Wulfften-Palthe) is a test that measures focus of attention and its fluctuation during the task. The subjects were required to examine 50 rows, and from the groups of 3/4/5 dots, they had to quickly and accurately allocate and strikethrough only the form of four dots (Figure 3). This test, tests psychometric parameters such as the total time for the entire test, the number of correct answers, omissions and errors. Psychogram represents the result of the test, with correct answers, errors, and omissions given in the function of time.

These traditional tests are all done in environments of the classroom. The laboratory of the Aeromedical institute where the psychological tests are done and the environment of the cockpit are not the same. Furthermore, the workload of the cockpit procedures includes demanding physical effort. The live training is where the personality pattern (confidence, making judgement under stress) comes in focus. The complete psychological selection rank depends on Personality inventory and Psychomotor testing, as well. The categorization given in this research follows only the pattern of Cognitive tests set data, described earlier. Therefore, indirectly, through the ranking list, it is possible to evaluate the efficiency of each candidate.

Due, to a transition from the analog cockpit in the propeller driven aircraft to a new aircraft (equipped with digital cockpit instruments) there is question whether these tests still cope with an existing training process, and subsequently the selection process. In this paper we evaluated the various advanced EM measures that enables understanding the changing importance of various regions on the display in order to pre-screen candidates even before they undergo first screening phase, or should it be used to compliment the psychological screening phase to eliminate low performing candidates from further screening.

### 2.2 Flight screening selection

**Figure 4:**
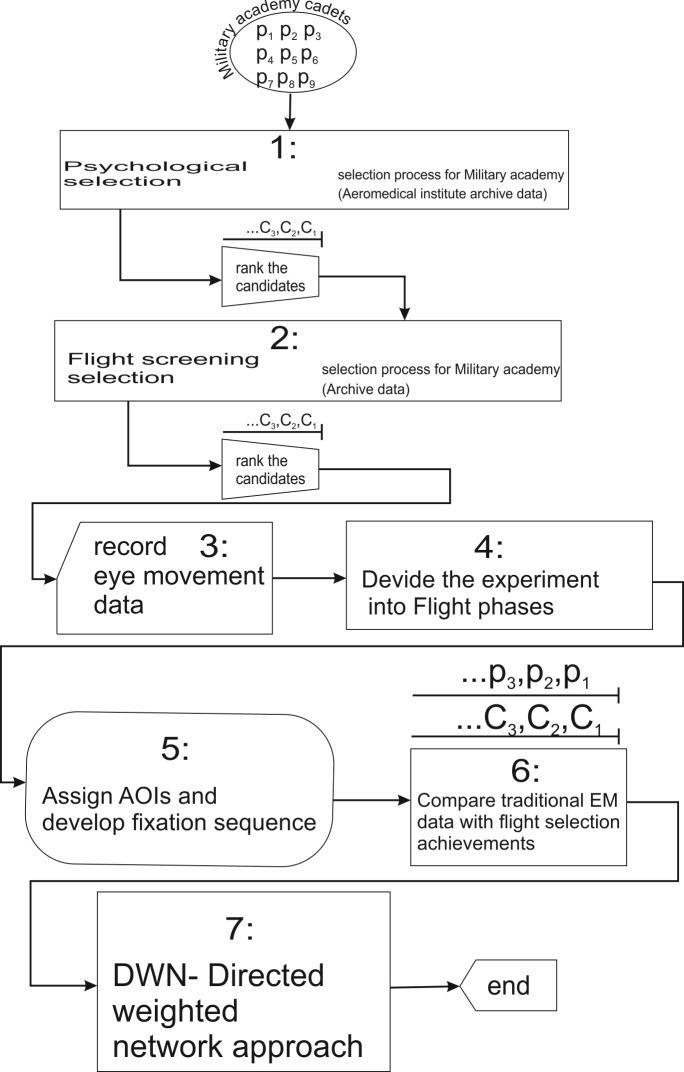
Flowchart of selection process and connection with eye movement research study.

According to the selection process (Figure 4), candidates who passed the psychological selection moved to the next step, the initial flight training, as the central part of the flight screening selection. The assessment during the initial flight training includes the grades (range 5 to 10) given by the flight instructors and flight examiners during ground training and in-flight evaluation. It consists of the candidate assessment regarding the senses and skills necessary for flying and piloting skills in the air.

There are five groups of senses and skills necessary for flying. For more precise evaluation, and according to their similarity, they are as follows: Working skills, Intellectual skills, Perceptive senses, Emotional senses, and Resulting skills^(2)^. The assessment of piloting techniques is in the air according to standard practices for monitoring, assessment, and evaluation^(20)^. It is done by a flight instructor in charge of that particular candidate and by an examiner during one VFR flight, with evaluation containing different elements.

The flight parameters (speed, altitude, bank angle) are predefined for every flight phase in the final flight that candidate flies with examiner^(2)^. Also, the allowed variation of these parameters is also defined, and for each grade there is defined range of variation from desired value^(2)^. The skills to maintain these parameters inside the predefined values, must be judged by the examiner, based on experience, thus inevitably resulting in potential drawbacks of traditional practice test for pilots regarding credibility^(21)^.

A concurrence between the EM analysis and their traditional counterparts (psychological, and flight selection) would further validate the use of low-cost eye tracking tools in the pilot selection process for digital avionics cockpits resulting in a significant cost saving.

### 2.3 Traditional EM measure and its application in pilot selection process

Table 1 shows the predictors of performance and scan behavior of both expert and novice pilots collected only, inside the flight simulation environment. Eye tracking has been a vital source of information about perception and cognition for more than 50 years^(22)^. It has been utilized to study a different number of topics, such as the patterns of fixations and saccades while reading text^(23)^ the workload of pilots during different phases of flight^(24)^ ^(25)^, the application of different scan patterns in flight training^(26),(16)^, the role of pilots' monitoring strategies in-flight performance(27) and the effectiveness of visual advertisements (28) and many others. Many previous studies on the terms of group selection dealt with the comparison of scan behavior characteristics of novice and professional pilots. Table 1 summarizes some of these studies.

We can see that both studies of Lijing, Hongpeng, Dayong, & Xiuly^(9)^ and and Comstock, Coates, & Kirby(29) investigated eye movement characteristics of pilots (beginners) during initial training process and in the research done by Yu, Wang, Li, Braithwaite, & Greaves(25)., the only participants were “18 qualified, mission-ready fighter pilots”.

In this research, eye tracking results consist of a total number of fixations inside all AOI's as in studies^(30),(31)^ and studies^(9),(12)^. This is a traditionally accepted approach. Research paper related to Eye-tracking data defines that "saccade from one AOI to the next is called a transition"^(8)^ also, the Saccade is referred as "a rapid eye movement from one fixation to another"^(8)^. The adopted scan-mode explained by Lijing & Lin^(21)^ considers that EM transition starts, i.e., from OC AOI then shifts to airspeed indicator AOI and then returns to OC. This sequence can start from any other AOI and constitute returning scan path or “revisit” with any different AOI. Lijing, Hongpeng, Dayong, & Xiuly^(9)^ explain the results of research of eye-movement indicators in correlation to the scanning technique and performance with experienced and novice pilots, particularly how saccade amplitude, saccade angle and time as eye fixation indicators correlate with flying skills. However, there was no indication whether these eye fixations indicators included the number of revisits. Mandal and Kang^(19)^ who investigated the Use of Eye Movement Data Visualization to Enhance Training of Air Traffic Controllers, searched the most appropriate approach to address the issues of frequent EF transitions between AOIs. They defined the Directed network visualization approach explained in Burch, Beck, Rasche, Blascheck, & Weiskopf^(32)^ as the most appropriate one. Indegree of a vertex (AOI) is the sum of all incoming weights to it from all other vertices in the network, where the weights are the amount of EF transitions between AOI pairs. When we carefully examine this, we can see that the EM transitions between AOI pair presume only the number of fixations.

Robinski and Stein^(12)^ who investigated how scanning techniques differ between experienced and inexperienced helicopter pilots indicated that there are “differences between TF (target fixations) as used by experienced pilots and Unintended TF used more likely by student pilots who will thus overlook crucial flight situation parameters.” Furthermore, they explain that the term Target fixations does not mean single fixation but total dwell time of the gaze towards particular target objects or instruments.

The necessity for the introduction of this term is the fact that eye track recordings analysis revealed that the same participants had a significant number of fixations inside some AOI, this is particularly true for outside cockpit (OC) fixations, but they were spread across vast area and not concentrated around one point or in some logical pattern.

In this experiment, we measured the number of revisits and the number of fixations, and the gaze duration was not measured. Comparing these two as a measurement of gaze pattern quality could be relevant since this is the comparison between three groups of novice pilots because it is more likely for them to make Unintended TF than a TF. On the other hand, just merely summing up the number of fixations in each of the AOI-s would not be a representation of a quality as we stated before.

Even some reference studies show that “even if there were fewer number of EFs or less durations, a target can be considered important if it plays a crucial role in the EM flow among multiple targets or acts as a bridge between two disconnected groups of targets.”^(19)^. The assumption is that the revisits that are counted when participant’s gaze leaves one AOI and makes the fixation inside any other AOI and then returns into the previous AOI show the following of proper scan-mode and thus quality, concerning the novice pilots in our case.

Most of the studies mentioned in the Table 1 dealt with scan characteristics of the qualified pilots and with comparison of those experienced and inexperienced, except in case of Lijing, Hongpeng, Dayong, & Xiuly^(9)^ Thus, findings of those studies can only be used in pilot selection process indirectly. We investigated the achievements of candidate pilots during Psychological and flight screening selection, and compare it with traditional and EM metric in simulated VFR flight environment. Furthermore, the results of the Network-based EM analysis are used to find the scan characteristics of different type of participants. The success in distinguishing between high, average and low-performance candidates could potentially help to reveal the implications of the introduction of the eye-tracking device on the selection process.

**Table 1:** Eye movement studies in Flight training

Nr.	Research	Group selection	Flight phases	Metrics
**1**	(Diaz et al. 2017)	*Expert pilots and cadets*	Final approach	fixation count, fixation duration
**2**	(Lijing et al. 2016)	*Novice and professional pilots*	Take-off	fixation count, fixation duration
**3**	(Yu et al. 2016)	*Expert and novice pilots (fighter pilots)*	Searching, pursuing, lock-on	saccade duration, pupil sizes, fixation duration
**4**	(Lijing et al. 2015)	*Novice cadet pilots (without any flying experience)*	Take-off, climb, flying, turn, approach, landing	fixation count, fixation duration, saccade amplitude, saccade angle, saccade time, average fixation time
**5**	(Yu et al.2014)	*Qualified, mission-ready fighter pilots*	preparation, aiming and release, break-away	fixation duration, pupil sizes, workload
**6**	(Robinski 2013)	*Experienced and inexperienced helicopter pilots*	Helicopter landing	TF number, performance, workload
**7**	(Kasarskis 2001)	*Expert and novice pilots*	Approach and landing	fixation count, fixation duration
**8**	(Comstock 1995)	*Pilots (beginners) during initial training process*	Level flight and turning	dwell times, the examination of sequences of dwells on the instruments

### 2.4 Network-based EM analysis

Mandal & Kang^(19)^ proposed the application of network-based EM analysis for dynamic tasks, where the various AOIs are shown as the vertices of a network and the EM transition among them is represented with directed edges between the vertices of the network. Developing the network-based visualization requires first creating the AOI transition matrix, which is tabular presentation of the eye fixation transitions occurring between various AOI pairs. Afterwards, this transition matrix information is used to develop the network representation, which visualizes the visual scanning strategy of the observer. For an in-depth study of the various steps involved in this process one should consult the paper by Mandal and Kang^(19)^. Furthermore, Mandal & Kang^(19)^ also introduced the application of three importance measures (adapted from network science domain) namely, Indegree, Closeness, and Betweenness, that helps to evaluate the importance of an AOI with respect to the visual scanning strategy of an observer^(33),(34)^. The Indegree of an AOI is defined as the amount direct attention received by it. Mathematically, it is defined as follows:

(i) $$I_{j}=\sum_{\begin{array}{l} k=1\\ k\neq j \end{array}}^{m}W_{kj}$$

Where, *w_kj_* is the number of EF transitions coming from *k^th^* AOI to the *j^th^* AOI and *m* is the total number of unique AOIs in AOI fixation sequence. In our case, number of AOIs is always four, thus m is a constant. The measure Closeness quantifies how important an AOI is in terms of its closeness to all other AOIs with respect to the visual scanning strategy of the observer. Note that this closeness measure should not be confused with physical proximity of the AOIs on the display, however, this should be interpreted as the closeness in the cognitive plane. Mathematically, Closeness of an AOI is defined as follows in Opsahl, Agneessens, & Skvoretz^(34)^

(ii) $$C_{j}=\sum_{\begin{array}{l} k=1\\ k\neq j \end{array}}^{m}\frac{1}{d_{jk}}$$

Where *d_jk_* is the minimum distance from the *j^th^* AOI to the *k^th^* AOI (if multiple paths exist) and *m* is the number of unique AOIs. The betweenness measure for the *j^th^* AOI is defined as follows^(34)^:

(iii) $$B_{j}=\sum_{k=1}^{m}\sum_{\begin{array}{l} l=1\\ j\neq l\neq k \end{array}}^{m}\frac{sp_{kl}^{j}}{sp_{kl}}$$

Where *sp_kl_* represents the total number of shortest paths (if multiple paths exist) from *k^th^* AOI to the *l^th^* AOI and the $sp_{kl}^{j}$ represents the number of such shortest paths which pass through the *j^th^* AOI.

## 3 Proposed Methodology

Figure 4 represents the flowchart of the pilot selection process and its connection with eye-movement research. Also, it represents the steps of the proposed methodology for analyzing, and comparison of the results from psychological, and flight screening stage of selection and with results of the recorded EM data.

### 3.1 Step 1. Collecting the results from the psychological selection

The input for this step are the results from the reasoning tests T2 and T5 tests, and Wulfften-Palthe (WP) (both paper and pencil-based tests) that are used to evaluate the candidates attention and perception abilities^(5),(6),(7)^.

Upon the completion of the process, the Aeromedical Institute, which is in charge of psychological selection, divides the candidates into three groups namely, high performance, average performance, and low performance. The complete psychological selection rank depends on Personality inventory and Psychomotor testing, as well. The categorization given in this research follows only the pattern of Cognitive tests set data, described earlier, and is a way of arbitrary classification. The cadets were ranked in terms of their total T score (complete sum/ achievements on three tests) they achieved during psychological selection.

### 3.2 Step 2. Collecting the results from the Flight screening

The ranking during the flight screening selection process is the result of the average performance of the three grades. Those are the grades obtained in the final flight with the examiner, and the two grades given by the flight instructor in charge of the participant (pilot candidate at that time). These grades are a result of continuous tracking of candidates’ development. All the candidates who have an average grade below 7.00 belong to low performance, between 7.00 and 7.50 and to average performance, and all those above 7.50 belong to high performance group^(2)^. In the present study, the total results, by all assessed categories during the initial flight training, were considered.

### 3.3 Step 3. Collecting the participant’s EM data

In this research, eye tracking results consist of a total number of fixations inside all AOI's as in studies^(30),(31)^ and also studies^(9),(12)^. This is a traditionally accepted approach. Furthermore, we decided to introduce measuring of revisits to illustrate the connection with the desired scan-mode for the participants. It consists of consecutive EM transitions between pair of AOIs. EM transition from one AOI to any other AOI and then returning EM transition to the previous AOI is one Revisit.

### 3.4 Step 4. Dividing the experiment into the time intervals (flight phases)

Time intervals were chosen based on task characteristics in event-based time intervals. This study investigated attention characteristics of groups of participants across the number of different flight tasks (phases of flight) in VFR flight scenario. Each participant took three 15-minute sorties. and the analysis between the time points inside these flights was chosen based on the researcher`s judgment.

### 3.5 Step 5. Defining the AOIs and developing AOI fixation sequence

We collected the eye movement data in four areas of interest (AOIs). The Areas of interest were: Airspeed Tape (AST); Altitude and Vertical Speed Tapes (AVST); Engine Instruments (EI) and Outside of Cockpit (OC). These AOIs were presumed as providers for the most critical information for the pilot's situational awareness (SA) while performing essential VFR flights. We created dynamic areas of interest, after recording flights and capturing eye fixations (EF) of participants. Dynamically interpolated rectangular of areas of interest (AOIs) frames and the analysis between the time points was chosen based on the researcher`s judgment defined in the step 4. Only those EFs falling within any of the AOI rectangle areas are considered for the AOI fixation sequence development.

### 3.6 Step 6. Comparison of traditional EM data with flight selection achievements

The primary goal of this step was to compare the results obtained by the use of an eye-tracking device with the results of psychological tests, which have been used for decades in the psychological selection of candidates and proven in practice. The successful comparison would serve as a predictor for the next stage, the flight screening. First, we investigated the use of the eye-tracking device as a useful prediction tool concerning the candidates' achievements at psychological tests, and the possible correlation with flight screening results (Figure 4). The result analysis will reveal the correlation of the EM metrics collected with an eye-tracking device with pilot selection process. The method of accomplishing that task is a statistical analysis of the collected data.

### 3.7 Step 7. Application of directed weighted network approach

A more in-depth study of visual scan paths of participants was done through network visualization applying steps for Directed Weighted Networks DWN representation. The tasks inside the simulation (flight phases) were arbitrary chosen (event-based) time intervals and the AOI sequences were develop from them. The study with DWN from Mandal, Kang, & Millan^(18)^ and modified Dynamic Network DNet approach introduced in Mandal & Kang^(19)^ were found to be appropriate. The steps applied in the network visualization process use as the input data from steps 3 to 5.

#### 3.7.1 Developing the AOI transition matrices for the selected time intervals

We transformed generated fixation sequence from the step 5 into an AOI transition matrix of the same size for each AOI sequence since the number of AOIs in our case is predetermined and always the same.

#### 3.7.2 Developing the DWN visualizations

We used the igraph package in R software for conversion of AOI transition matrices to graphs^(35)^. Representation of the network was done by adopting the design principles suggested in Mandal et al. (2016) with some modifications. There is the difference with suggested principles of the color of a vertex. Based on the RGB palette of the RColorBrewer package in R software, the red color means low EF duration, orange color represents higher and yellow color the highest EF duration occurring on the associated AOI.

#### 3.7.3 Calculate and visualize target (or AOI) importance measures

New network-related metrics is calculated instead of the previous EM metrics. Three node related centrality measures are proposed by Newman^(33)^ Opsahl, Agneessens, & Skvoretz^(34)^ namely; indegree, closeness and betweenness.

The last step of network visualization process represents the normalization process for all three target importance measures. The distance normalization process presents calculation of how far away a given importance measurement of an AOI is from the maximum value observed for that measurement within the time interval. Bar plot representation of the normalize relative importance measures of four AOIs during five flight phases for each of three participants helps in comparing a single importance measure.

Distance normalization which is used in this paper refers to calculating how far away a given importance measurement of an AOI is from the maximum value observed for that measurement within a time interval (flight phase). The distance normalized measure for the *j_th_* AOI for time interval t is calculated as:

(iv) $${\tilde{\varphi }}_{j}(t)=\frac{\varphi_{j}(t)-\min_{j}\varphi_{j}(t)}{\max_{j}\varphi_{j}(t)-\min_{j}\varphi_{j}(t)}$$

Where, *max_j_ φ*(*t*), *min_j_ φ*(*t*) and ${\tilde{\varphi }}_{j}(t)$ is the maximum, minimum and distance normalized value of the measure *φ_j_* (*t*) respectively $\left(0\leq {\tilde{\varphi }}_{j}(t)\leq 1\right)$.

## 4 Experiment

### 4.1 Scenario

We collected the eye movement data in four areas of interest (AOIs). The Areas of interest were: Airspeed Tape (AST); Altitude and Vertical Speed Tapes (AVST); Engine Instruments (EI) and Outside of Cockpit (OC). These AOIs were presumed as providers for the most critical information for the pilot's situational awareness (SA) while performing essential VFR flights. Those data and crosschecking patterns are recommended or defined in the official manuals used by candidates and trainees^(36),(37)^. No matter whether there are moving cursors in circular or semicircular areas, Moving-Tapes (linear moving scale) with or without trend information, changing digits, or changing colors and symbology of primary flight display (PFD), Flight deck itself, with its instrument panel (in traditional T - layout or modern glass cockpit), represents the source of dynamic stimuli for observer^(17)^. Components of the glass cockpit such as Air Speed Tape (AST); Altitude and Vertical Speed Tapes (AVST); Engine Instruments (EI) are the ones that offer dynamic stimuli. Outside the cockpit (OC) area, is a part of the screen viewed during the flight where changes between the projection of the cockpit frame onto the horizon and the horizon layout itself occur. This area is especially essential for the training of beginner pilots since instructors teach them in scan-mode of attention to "OC, airspeed indicator, OC, attitude indicator, OC, altimeter”^(21)^. We created dynamic areas of interest, after recording flights and capturing eye fixations (EF) of participants. Time intervals were chosen based on task characteristics in event-based time intervals. This study investigated attention characteristics of groups of participants across the number of different flight tasks (phases of flight) in VFR flight scenario. Dynamically interpolated rectangular of AOIs frames and the analysis between the time points was chosen based on the researcher`s judgment.

In the eye movement research related to Pilot's attention allocation by Anders^(38)^ and visual scanning patterns done by Rudi, Kiefer, & Raubal^(17)^, Primary Flight Display (PFD) and Navigation Display (ND) are areas that receive the highest visual attention. The eye-tracking tool used in this experiment depicted airspeed indicator and altitude and vertical speed tapes which are on the far left and right side of the Garmin G500 Primary flight display PFD. Earlier findings by Anders^(38)^ proved that speed indicator with 7.8%, altitude indicator with 7.6% and vertical speed indicator with 3.3% are the areas that received the highest visual attention inside PFD after Artificial Horizon (AH) with 10.5%. There are many other information fields besides those two, (i.e., Roll scale, Slip/Skid indicator) and the recordings showed that the participants have tried to fixate on them. An airplane with a traditional flight panel (T-layout), where all the information was "scattered" amongst different instruments, was used for the ab-initio training. Concerning that, only AST and AVST were taken into account since these two provide the most crucial information inside the cockpit for VFR flights.

On the other hand, during the ab-initio training, the participants were taught in the manner of the previously mentioned scan mode^(36),(21),(37)^. By respecting that rationale, the information about the changes of the bank angle is firstly registered as differences between the cockpit frame projection onto the horizon seen in Outside of Cockpit (OC) area, and later from Artificial horizon (AH). For that reason, participants use OC to correct the bank angle and disregard the Artificial horizon (AH), accordingly. Each participant took three 15-minute sorties. The number of fixations and revisits for each of the five flight tasks across three sorties was their average performance.

### 4.2 Tasks

The candidates were required to undergo five different tasks while flying the aircraft under VFR conditions. These tasks are: 1) Take-off, 2) Climb, 3) Strait-and-level-flight, 4) Turn, and 5) Final approach and landing

### 4.3 Participants

A total of nine trainees participated in longitudinal research. All of them had completed ab-initio flight training and had limited aeronautical knowledge and flight experience based on screening flights only. Total flight hours per trainee were 10 hours on a light piston-engine aircraft that belonged to the utility category^(10)^. During initial flight training, the participants/trainees were given lectures on proper attention distribution-pattern (scan-mode) in different VFR flight phases. All of the participants were males ranging from 20 to 24 years (M=22 years, SD=1.18 years).

### 4.4 Apparatus

#### 4.4.1 Hardware

##### 4.4.1.1 Flight simulator

**Figure 5:**
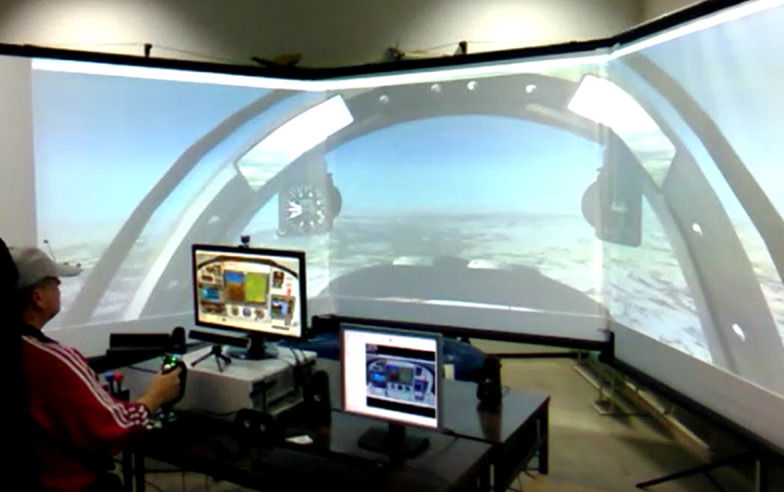
Simulator setup with eye-tracking tool equipment.

We created the flight simulator by use of COTS (Commercial-Off-The-Shelf) components, including both hardware and software, based on Microsoft Flight Simulator X (FSX). This software package installation was on the hardware configuration that was commercially available at the time of the research study^(39)^. A specific add-on possesses the characteristics of the Lasta military trainer with its digital cockpit. Figure 5 shows an image of the simulator setup.

##### 4.4.1.2 Eye Tracking Tools

We have used GP3 Desktop Eye Tracker (sampling rate 60 Hz, visual angle accuracy 0.5o – 1°) to record the pilot's eye movements^(40)^.

#### 4.4.2 Software

GP3 Desktop Eye Tracker software exports two types of EM data in “.csv” Excel datasheets. EM data concerning the fixations consists of horizontal and vertical coordinates (“position of gaze x” and “position of gaze y”) gaze time (starting from the beginning of recording), gaze duration, and gaze ID. The second file is a Data Summary report with AOI names and the associated number of fixations and revisits. Also, there is a time of the start of the AOI sequence for each of the five AOI sequences (flight phases).

Microsoft Flight Simulator X (FSX) Microsoft Flight Simulator X, also known as FSX, is a 2006 flight simulation computer game originally developed and published by Microsoft for Microsoft Windows. Flight Simulator X was released in three editions: Standard, Deluxe, and later Gold. The Deluxe Edition features 24 aircraft compared to 18 in the Standard Edition.

Previous, long-term process, of the research team Vlačić, Knežević, & Milutinović^(39)^, to simulate the specific piston-engine military trainer, which was not involved in the FSX package generated an appropriate add-on of Lasta training aircraft. The add-on replicated both the aerodynamic model and the new instrument panel of the mentioned airplane. We used the igraph package developed by Csardi & Nepusz^(35)^ in R software for conversion of AOI transition matrices to graphs, and for calculation of network-based advanced EM measures.

### 4.5 Data analysis

#### 4.5.1 Traditional EM metric data analysis

The primary goal was to compare the results obtained by the use of an eye-tracking device presented in the form of number of fixations and number of revisits with the results of psychological tests. These tests have been used for decades in the psychological selection of candidates and proven in practice. The successful comparison could potentially serve as a predictor for the next stage, the flight screening. First, we investigated the use of the eye-tracking device as a useful prediction tool concerning the candidates' achievements at psychological tests. Later, the possible correlation with flight screening results will be investigated. These actions are presented in Flowchart inside step 6 of the research study (Figure 4).

There are several groups of results or the candidate’s achievements obtained through different types of trials or tests, and they create the research database. Those are:

- Psychological tests (Figure 8, and Table 4– We named this step of grouping candidates using psychological test scores as ‘psychological selection stream’)
- Eye-tracking tests ( Figure 10, Figure 11, Figure 12, Table 3 and Table 4)
- Flight-screening results (Figure 9 and Table 5 - We named this step of grouping candidates using flight screening scores as ‘flight screening selection stream’).

There is only one common attribute for normalization of this database. The candidate`s achievement or rank at some of the trials is the one that permits it to be queried and manipulated.

In the first phase, naming the test subjects Participants (P1, P2) was done, according to their results in the early stage of selection. The key is {Psychological selection rank- participant ID}. The participant who scored the most points in psychological screening was named P1 (Table 3). Accordingly, all other participants were named.

Concerning our study, the selection process has already been finished (Figure 4). The Military academy cadets who had completed ab-initio (flight screening) flight training (Figure 4) and had limited aeronautical knowledge and flight experience based on the screening flights only were participants in this research. Total flight hours per trainee were 10 hours on a light piston-engine aircraft. The results of psychological tests and flight screening, were already available, making this research as some way of post-hoc selection or analysis.

Conditionally the research done inside step 6 of Proposed methodology (Figure 4), was presented in data analysis into two separate streams. The first stream is conditionally more psychologically oriented and compares the participant`s previous results on the psychological selection with the eye-tracking device results. Since this step of the pilot selection process precedes flight screening in the timeline it will be a discussed first, and will be named Psychological selection stream.

The second one, named Flight screening selection stream, compares participant`s achievements on flight screening with the results of the recorded EM metrics during simulated flight scenario. It should be noted that the candidates who passed both psychological and flight screening stage of selection and moved the next step, became Military academy cadets. We used only their achievements for comparisons.

#### 4.5.2 Network based data analysis

GP3 Desktop Eye Tracker exports two types of EM data in “.csv” Excel datasheets. EM data concerning the fixations consists of horizontal and vertical coordinates (“position of gaze x” and “position of gaze y”) gaze time (starting from the beginning of recording), gaze duration, and gaze ID. The second file is a Data Summary report with AOI names and the associated number of fixations and revisits. Also, there is a time of the start of the AOI sequence for each of the five AOI sequences (flight phases). For extracting gazes within defined AOIs coordinates, we used the start time of AOI sequence (phase of flight) from Data Summary report. Since we choose event-based intervals, of known duration, we selected an array of gaze IDs (rows of Excel sheet) within that interval. We analyzed Each AOI sequence with the use of data Filter tools in Excel. Filtering data columns with coordinates of AOIs (within specified rows) for each of them, gave us fixation sequence. The final fixation sequence is a collapsed form of raw fixation sequence, same as Mandal & Kang^(19)^. Namely, Multiple consecutive fixations of the same AOI collapsed into a single fixation.

We transformed generated fixation sequence to an AOI transition matrix (sample for participant P9 given in Table 2) of the same size for each AOI sequence since the number of AOIs in our case is predetermined and always the same.

**Table 2:** AOI transition matrix developed from the AOI fixation sequence for time interval of Straight-and-level phase of flight for participant P9

**FROM AOI**	**TO AOI**			
	AST	AVST	EI	OC
**AST**	0	1	2	6
**AVST**	1	0	1	8
**EI**	1	0	0	3
**OC**	7	8	2	0

**Figure 6:**
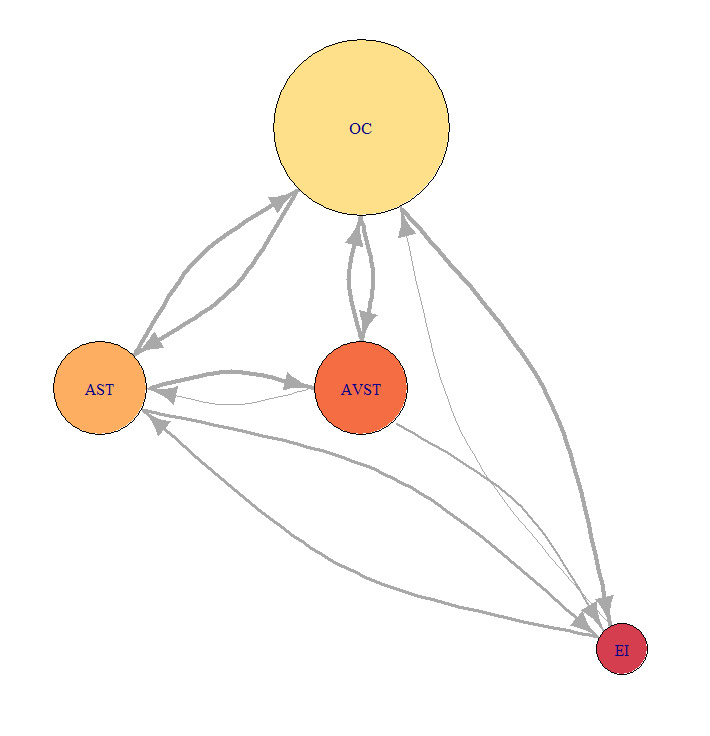
Sample of static network visualization of AOI transition matrix from Table 2.

Representation of the network was done by adopting the design principles suggested in Mandal, Kang, & Millan, 2016^(18)^ with some modifications. We used the igraph package in R software for conversion of AOI transition matrices to graphs^(35)^. There is the difference with suggested principles of the color of a vertex. Based on the RGB palette of the RColorBrewer package in R software, the red color means low EF duration, orange color means higher and yellow color means the highest EF duration occurring on the associated AOI. Network’s vertex radius size is proportional to the number of EF received by the corresponding AOI. Also, the thickness of an edge between a vertex pair is proportional to the number of EF transitions occurring between vertices in the edge’s direction same as Mandal, Kang, & Millan^(18)^. Therefore, for the example given in Table 2 we have static network presentation as in Figure 6.

**Figure 7:**
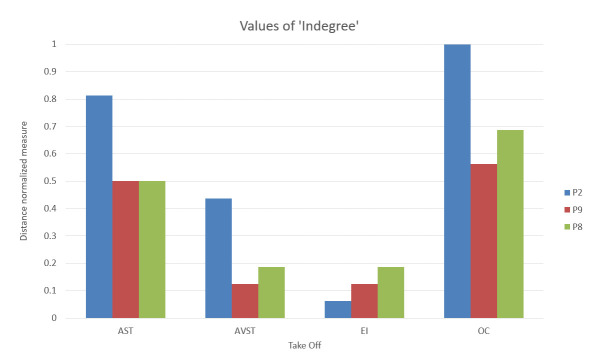
Bar plot shows the relative indegree values for defined AOIs among three Participants act as representatives of their groups. The color scheme applied in previous bar plots (blue- High-performance participants, Red- Average-performance, and Green- Low-performance)

For this study, we chosen three characteristic participants. Each participant belonged to one of the previously defined groups. We developed the static network for each of the five flight phases, for each of the three participants. Calculation of AOI importance measures was done using equations (i), (ii), (iii) with the igraph package in R software and also normalized for better comparison. Distance normalized measure of indegree of sample bar plot, for defined AOI, and participants P2, P9 and P8 are presented in (Figure 7).

## 5 Results

### 5.1 Psychological selection stream analysis

**Figure 8:**
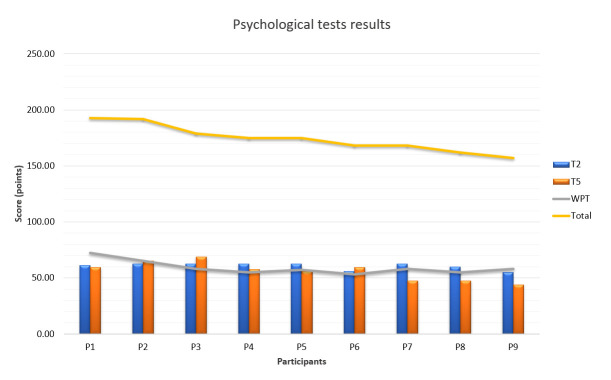
Results of the psychological selection stream analysis.

Figure 8 shows the results of Tests T2 and T5 (measuring visual perception) and WP (Wulfften-Palthe) test (measuring the distribution of focused attention). The cadets were ranked in terms of their total T score (complete sum/ achievements on three tests) they achieved during psychological selection. The yellow trend line in Figure 8 shows the T score value. The overall ranking of individual participants is shown in Table 3.

Upon the completion of the process, the Aeromedical Institute, which is in charge of psychological selection, divides the candidates into three groups namely, high performance, average performance, and low performance. The complete psychological selection rank depends on Personality inventory and Psychomotor testing, as well. The categorization given in this research follows only the pattern of Cognitive tests set data, described earlier, and is a way of arbitrary classification. In this study, we divided participants into three categories based on the total score calculating 67^th^ percent and 33^rd^ percent values from the sample of total scores in Table 3, since the sample distribution was not normal.

**Table 3:** Ranking of all cadets for the psychological selection test.

**Participant**	**P1**	**P2**	**P3**	**P4**	**P5**	**P6**	**P7**	**P8**	**P9**
**Total score**	192.5	192	179	175	175	168	168	162	157
**Psychological selection rank**	1	2	3	4	5	6	7	8	9

The division was made using equations (v) and (vi) shown below:

(v) $$67^{th}\text{percent}=\min+0.67*(\max-\min)..$$

(vi) $$33^{\text{rd}}\text{percent}=\min+0.33*(\max-\min)..$$

Where, *max max * and *min* represents the maximum and minimum value of the T score respectively. Inserting the respective value in equation (4) and 5) we get that, 67^th^ percent=157+ 0.67* (192.5 – 157) =180.758, and the 33^rd^ percent =2+ 0.33* (192.5 – 157) = 168.715.

Table 4 shows the candidates categorized into three performance groups using the above-mentioned threshold values. Note that the low performance candidates are the ones who actually passed the psychological selection, others who fail to pass were not considered. We named this step of grouping candidates using the psychological test scores as ‘psychological selection stream’.

**Table 4:** Categorization of candidates into three performance groups for psychological selection test.

**Rank**	**Participant**	**Group**
**1**	P1	High performance
**2**	P2	
**3**	P3	Average performance
**4**	P4	
**5**	P5	
**6**	P6	Low performance
**7**	P7	
**8**	P8	
**9**	P9	

After the candidates are grouped into different performance groups, we further analyzed their visual scanning patterns to find whether participants, belonging to different groups, exhibit different visual scanning strategy or not.

### 5.2 Flight selection stream analysis

**Figure 9:**
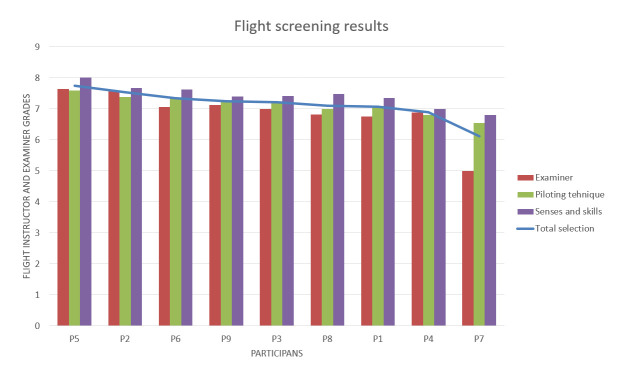
Results of the Flight screening stage of selection.

Figure 9 represents the scores for the various participants for the ‘flight screening phase’. The blue line shows the average of all screening grades. Table 5 shows the ranking of the participants based on their scores in this stage of pilot selection process.

The ranking during the flight screening selection process is the result of the average performance of the three grades. Those are the grades obtained in the final flight with the examiner, and the two grades given by the flight instructor in charge of the participant (pilot candidate at that time). These grades are a result of continuous tracking of candidates’ development. All the candidates who have an average grade below 7.00 belonged to low performance, between 7.00-7.50 average performance, and all those above 7.50 belonged to high performance group^(2)^ Table 5 also enlist the group each participant belongs to.

We named this step of grouping candidates using the psychological test scores as ‘psychological selection stream’.

Comparing the Table 4 and Table 5, it is quite clear that the Participants P2, P3, and P7 are the only ones that have kept their original groups. Participant P2 even held its original rank. A conclusion could be that psychological tests are by no means a ranking predictor for flight performance. The rank of the participant P2 might be just a coincidence. A situation like this was expected, due to an environmental factor (ergonomics of the cockpit, noise, communication with ATC) that affected most of the participants during flight screening. As a result, their ranks (represented in the Tables 4 and 5) are not the same.

**Table 5:** Results of the flight screening selection

**Flight selection Rank**	**Participant**	**Flight selection grades (total)**	**Group**
**1**	P5	7.75	high performance
**2**	P2	7.74	
**3**	P6	7.35	average performance
**4**	P9	7.26	
**5**	P3	7.21	
**6**	P8	7.10	
**7**	P1	7.07	
**8**	P4	6.89	low performance
**9**	P7	6.11	

### 5.3 Traditional EM metric result analysis

**Figure 10:**
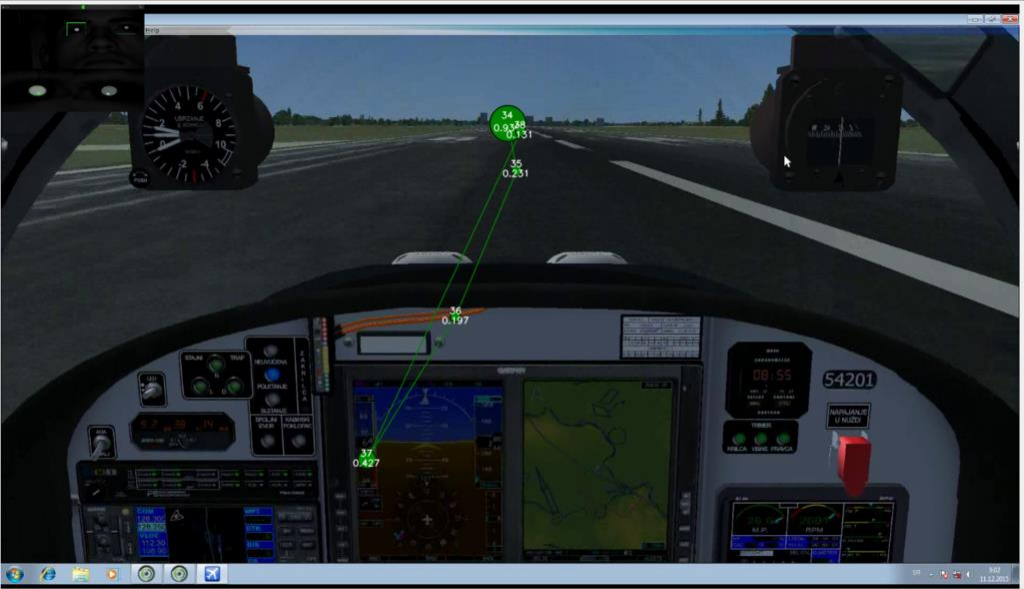
Recording of participant P5 Take off phase. Gaze plot of 3 seconds duration. Fixation (Green circles) of evidently smaller radius and thus shorter duration. Saccades (transitions) from AOI ‘AST’ to AOI ‘OC’ present.

Recording of the traditional EM metrics showed that some of the participants displayed some basic flight skills they had previously adopted. The adopted scan-mode explained by Lijing & Lin^(21)^ considers that EM transition starts, i.e., from OC AOI then shifts to airspeed indicator AOI and then returns to OC (Figure 10).

**Figure 11:**
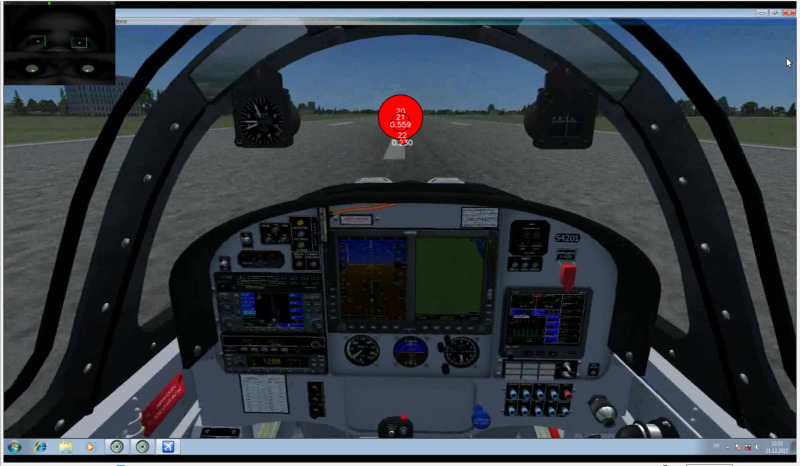
Recording of participant P7 Take off phase. Gaze plot of 3 seconds duration. Fixation (Red circles) of evidently larger radius and thus longer duration. Saccades from AOI ‘AST’ to AOI ‘OC’ (or vice versa) not present.

Figure 10 represents an illustration of the desired scan-mode. The difference between one participant who followed the desired scan-mode during take-off shown in Figure 10 (green color circles) and made returning transitions with fixation of shorter duration (represented with smaller radius) and the participant shown in Figure 11(red color circles) who made only consecutive gaze points outside the cockpit and made fixation of longer duration represented with larger radius illustrates the importance of revisits.

The recording of the Participant P7 shows that the desired scan mode was not adopted, and that he was making Unintended TF inside AOI ‘OC’, previously explained in Robinski and Stein^(12)^ and consequently great number of fixations. Taking into account only the number of fixations could lead us to assumption that he had greater quality of scan pattern, which is not the case. Figure 11 shows that the circle radiuses that represent the gaze duration are grater, and the EM transition was not shifted from OC to speed indicator.

**Figure 12:**
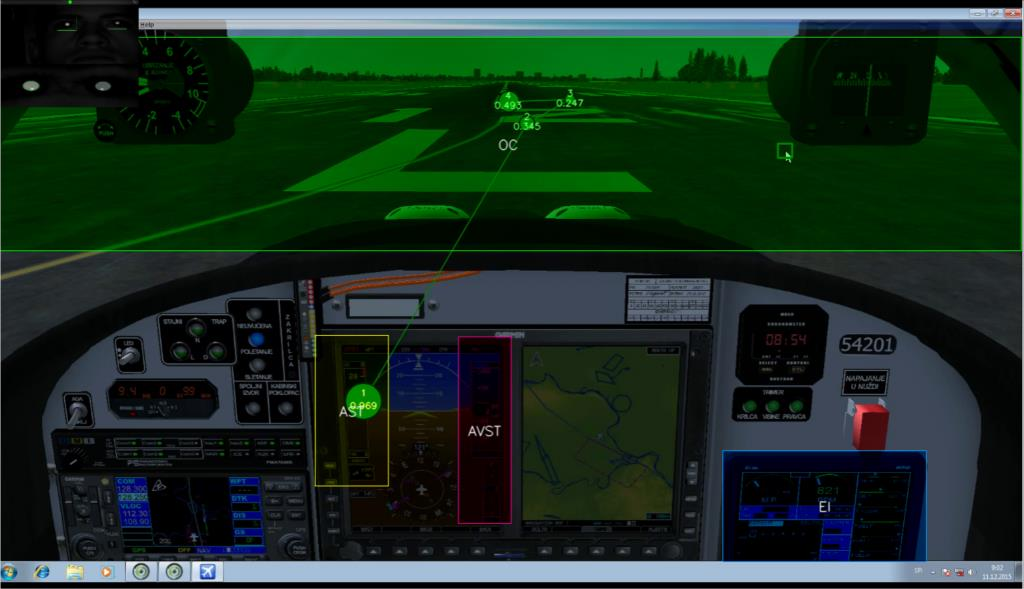
Position of Areas of interest (AOIs.) Fixations No26 to No32, during 3 second period. Gaze point analyzer was set up to refresh display with gazes on every 3 seconds. Participant P5 example.

The Areas of interest were: Airspeed Tape (AST); Altitude and Vertical Speed Tapes (AVST); Engine Instruments (EI) and Outside of Cockpit (OC). The defined AOIs (Figure 12) were presumed as providers for the most critical information for the pilot's situational awareness (SA) while performing essential VFR flight (see 4.1).

Comparison of the traditional EM data with the achievements of the participants in the psychological selection was done using the groups previously defined in Table 4. (Categorization of candidates into three performance groups for psychological selection test).

**Figure 13:**
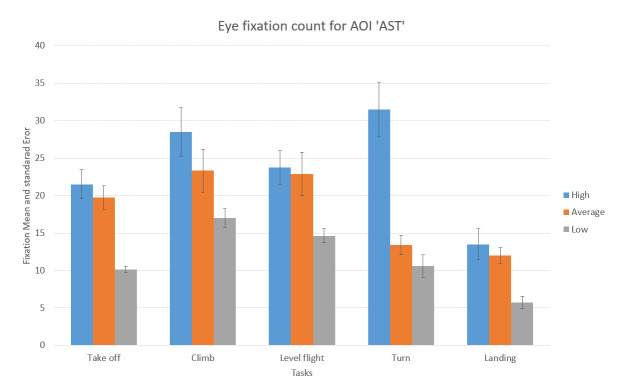
Number of eye fixations inside AOI ‘AST’ for five flight phases for the psychological selection stream with associated standard errors.

In Figure 13, we can see the result, which represents the mean number of fixations inside AST AOI across the five flight phases, with associated standard errors, taken from recordings of three sorties that each participant carried out.

**Figure 14:**
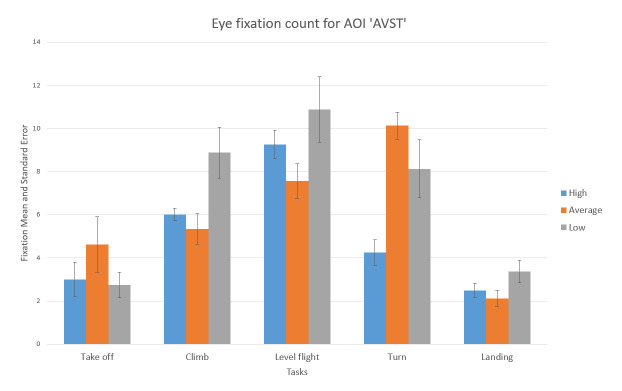
Number of fixations AOI ‘AVST’ for five flight phases for the psychological selection stream with associated Standard Errors.

For the AOI AST the high-performance participants had a greater number of eye fixations compared to other two groups (see Figure 13). Unlike this, for the AOI AVST, high-performance participants had a smaller number of eye fixations count then average, and low-performance ones, during all five phases (Figure 14). Low-performance had the highest results, in the number of fixations inside AOI AVST with the exception of ‘Take-off’ and ‘Turn’ phase where the average participants had the most in rest of the three phases (see Figure 14).

**Figure 15:**
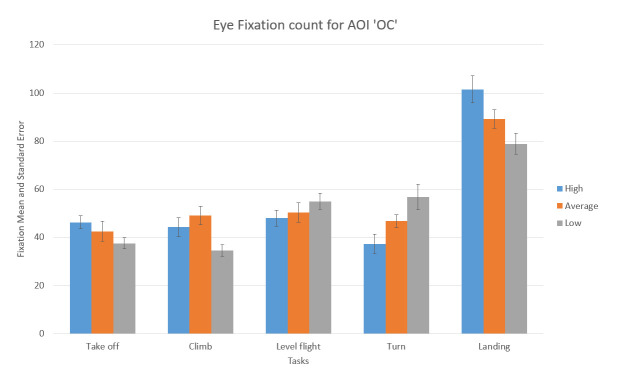
Number of fixations and standard errors AOI ‘AST’ for five flight phases for psychological selection stream.

In case of the AOI OC, high-performance participants had the greater number of fixations during Take off and Landing phase and slightly a smaller number of fixations during the other phases than average and low-performance participants (Figure 15).

We can notice that all participants displayed some basic flight skills they had previously adopted since the highest number of fixations made across all five phases for all three groups was during landing phase inside AOI “Outside cockpit” (Figure 15).

One-way ANOVA revealed significant differences between groups only in the number of fixations inside AST AOI (F(3. 21299) = 10.03922 p<0.01). Pairwise t-test between groups revealed significant difference between High-performance participants (M =23.582,SD=7.42) and Low-performance participants (M = 28.037, SD = 5.16), (t(4.72) = 2.048 p < 0.01) and High-performance participants( M = 23.582, SD = 7.42) and Average-performance participants (M =17.016, SD =7.44),(t(2.14) = 2.068 p < 0.05). High-performance participants had a greater number of fixations inside (OC) AOI than Average-performance, and Low-performance participants did. All other AOIs results did not show any statistically significant difference between the groups.

**Figure 16:**
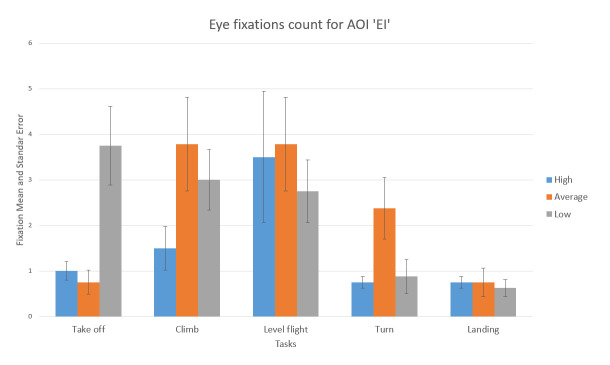
Number of fixations AOI ‘EI’ for five flight phases for psychological selection stream

The number of fixations inside EI AOI is insignificant in absolute and relative number. It is represented in Figure 16. This fact was another confirmation that the participants were untrained beginner pilots (skilled pilots would pay more attention to engine parameters). This information can be a future reference for the evaluation of sorties in the flight simulator.

In case of number of Revisits One-way ANOVA, again, revealed significant differences between groups in number of revisits inside AST AOI (F(3.2199) = 6.7093, p<0.01). Separate independent T tests between groups revealed significant difference between High-performance participants (M=15.532, SD=5.11) and Low-performance participants (M=9.0245, SD=4.24) (t[3.566]=2.048 p<0.01) and no significant difference between High-performance participants (M=15.532, SD=5.11) and Average-performance participants (M=12.9046; SD=4.85), (t(1.24405)=2.0686, p= 0.2260). The comparison between Average-performance participants and Low-performance participants revealed that the Average one with (M=12.9046, SD=4.85), had statistically significant greater number of revisits then Low-performance participants (M=9.0245, SD=4.24), (t[2.4428]=2.0345 p<0.02).

**Figure 17:**
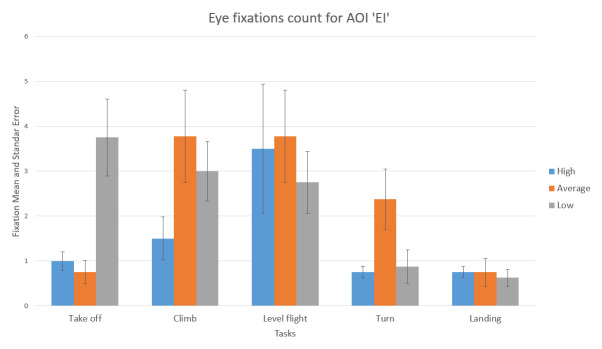
Number of revisits AOI ‘AST” for five flight phases for psychological selection stream with associated Standard errors.

Figure 17 shows that Average-performance participants had some more revisits than High-performance did during climb. It is an additional confirmation of active visual scanning pattern by High-performance participants, regarding the results they had. A study done by Katoh^(41)^ proposed (for landing phase) attention of both instruments and the outside cockpit confirms that the most active scanning pattern should exist during Landing phase.

**Figure 18:**
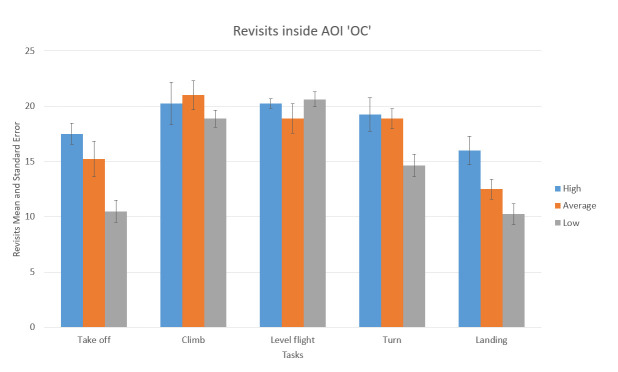
Number of revisits AOI ‘OC’ for five flight phases for psychological selection stream with associated Standard errors.

The use of recorded number of revisits shown in Figure 17 and Figure 18, and the fact that ranking of the participants across all flight phases in both cases, the number of fixations, (Figure 13 and Figure 15) and the number of revisits (Figure 17 and Figure 18) is descending from high to low, prevailed to conclude that the High-performance participants were making Target fixations and had more agile scanning pattern.

Accordingly, the rank of the participants established during psychological selection correlates with the eye-tracking device results, regarding the number of fixations and revisits.

**Figure 19:**
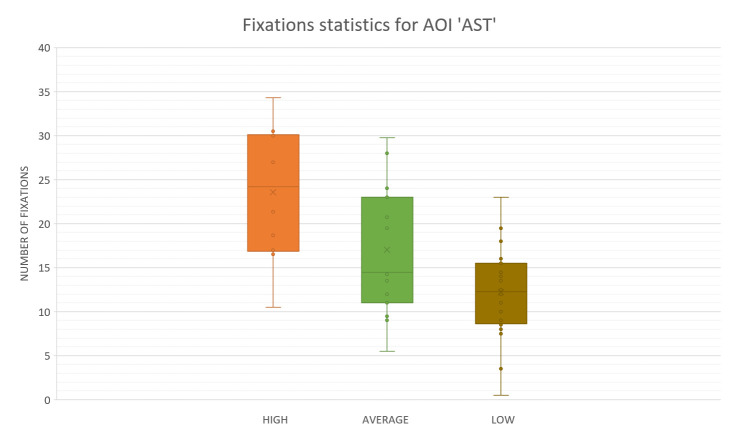
Box plot for the number of fixations inside AOI ‘AST’.

Results presented in the form of box and whiskers in Figure 19 confirm that the High-performance participants had the statistically most significant number of fixations inside AST AOI.

**Figure 20:**
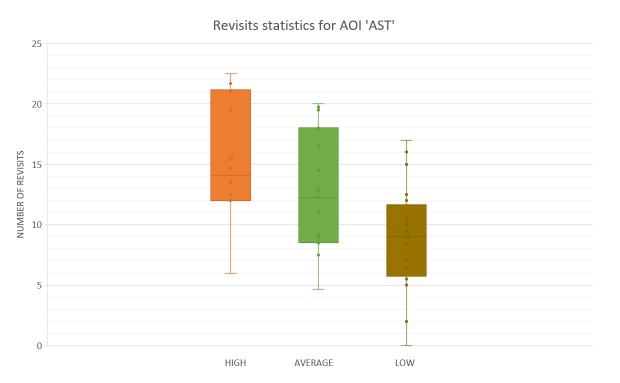
Box plot for the number of revisits inside AOI ‘AST’.

Concerning the statistics of the revisits inside AOI AST Figure 20 shows the results, and rank between the three groups of participants.

In the next step of the comparison, the EM data were compared with the ranks, and the groups of the participants established according to the flight screening selection results (Table 5 Results of the flight screening selection). In the same manner as in the psychological selection stream when we tried to analyze it statistically, One-way ANOVA applied to the number of fixations reported no significant statistical difference between the groups, except in the case of EI AOI, which is not so important AOI during VFR flights. The situation with statistical confirmations of difference between the participants in the number of revisits inside (OC) AOI, is almost identical with the fixation count. Once again, the correlation between the flight screening results and the laboratory environment tests (EM metrics in this case) was not confirmed. This was also case with the use of psychological tests as a predictors of flight screening results (see 5.2).

### 5.4 Network based EM metrics results analysis

Previous section showed that the use of traditional EM metrics (number of fixations and revisits) as a predictor for the Flight selection process of Military academy cadets failed to show any correlation with Flight selection rankings of participants. Furthermore, there was no certain evidence of saccade strategy existence, and the difference between test subjects; thus, more refined approach was needed.

Representation of the network was done by adopting the design principles suggested in Mandal & Kang^(19)^. Since the input for the fixation sequence was the number of the EFs we provided the table with average number of fixation and chose the three characteristic participants.

These three participants are representatives of their groups (High-performance, Average-performance, and Low-performance) according to their overall number of fixations. Using equations (v) and (vi), we will have a situation given in Table 6.

**Table 6:**
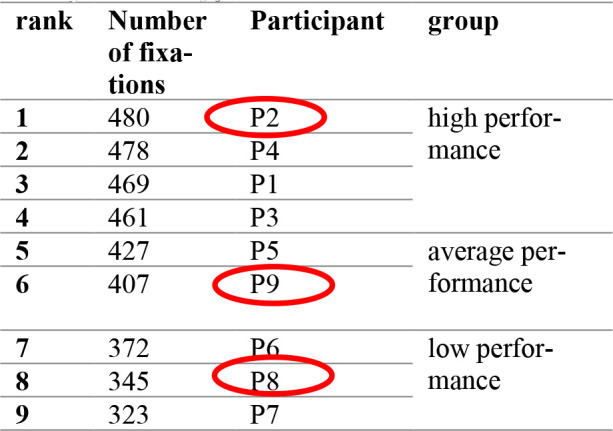
Average number of fixations, overall achievements

According to Table 6 participant P8 is in the Low-performance group. He was also in the Low-performance group in the Psychological selection (We named this step of grouping candidates using the psychological test scores as ‘psychological selection stream’ Table 4) and even his average performance grade on the Flight screening selection was close to 7.00 (Low performance) Table 5. The other two participants, P2 who belongs to High-performance group, and P9 who belongs to Average-performance group, (Table 5) both belonged to the same groups during Flight screening selection, respectively (Table 5).

**Figure 21:**
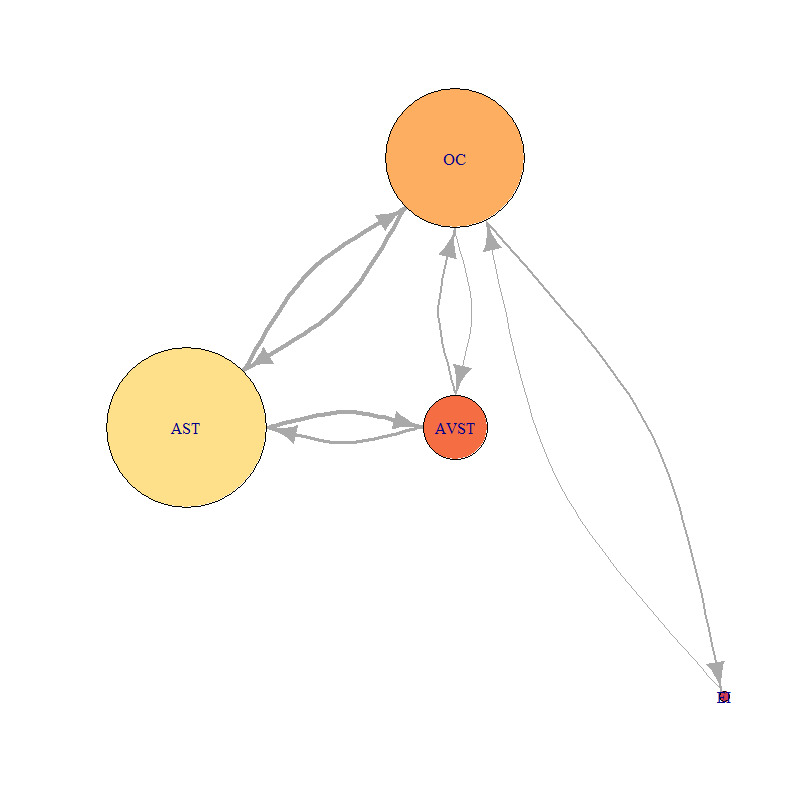
Network visualization of EM data for participant P2, during Climbing.

**Figure 22:**
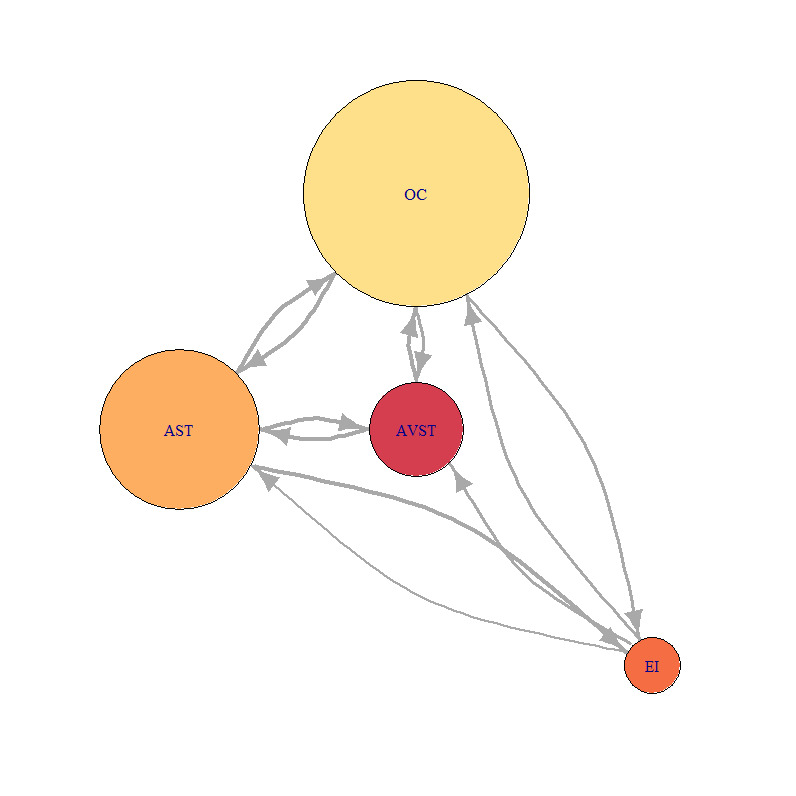
Network visualization of EM data for participant P9 during Climbing.

**Figure 23:**
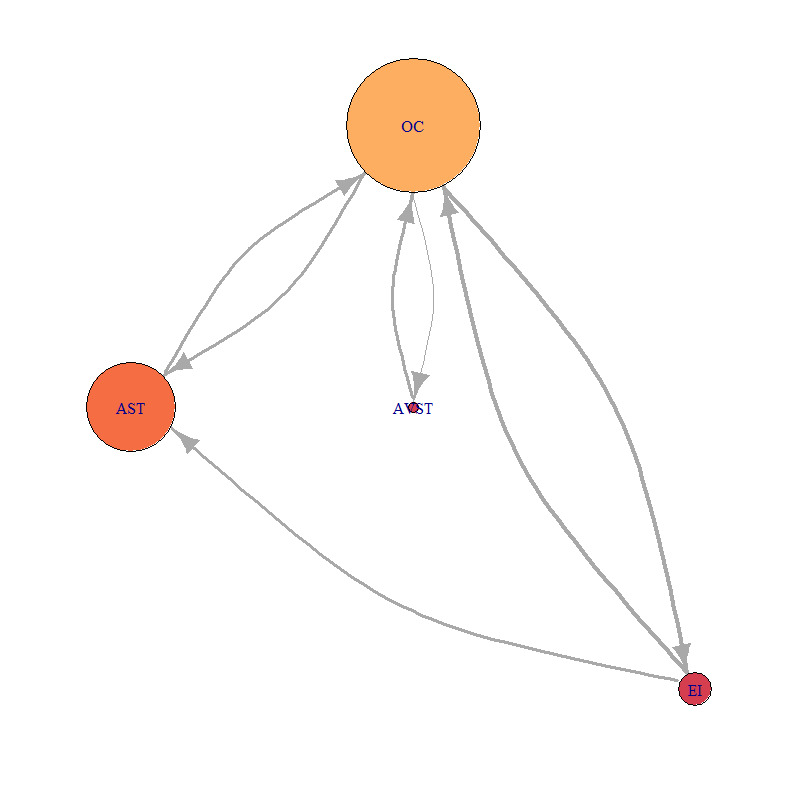
Network visualization of EM data for participant P8 during Climbing.

Figure 21, Figure 22 and Figure 23 represent network visualization of the EM data for three participants during Climb phase of flight, for all four AOIs. In Figure 21 AOI AST is the primary one for participant P2 since it has most EF numbers (circle size) and longest EF duration (circle color), closely followed by OC AOI. AVST AOI is substantially less important, whilst EI AOI has almost no importance.

For the Average-performance participant P9 (Figure 22) AOI OC is the primary one. Two AOIs, AST and AVST are considerably less important. AVST AOI has a shorter EF duration then EI AOI on the other hand, AST has longer EF durations and greater EF numbers. Unlike in the previous case, AOI EI possess some importance for participant P9. Figure 23 shows that primary AOI for participant P8 was AOI OC. Compared with previous cases this is the least important “primary AOI” since it has least EF numbers (circle size), and shortest EF durations (circle color). In this case, AOI AST is of secondary importance, with two other AOIs disregarded. Figure 21 through 23 also shows that there is a difference in the number of EF transition between some AOIs and that some connection between AOIs does not exist for some participants.

**Figure 24a:**
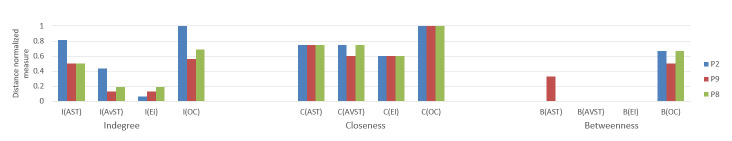
Distance normalized measure values for AOIs during Take-off flight phase.

**Figure 24b:**
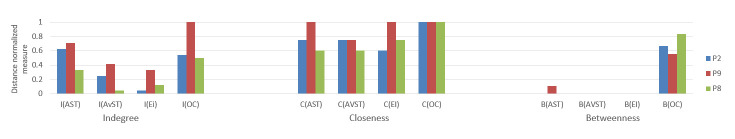
Distance normalized measure values for AOIs during Climb flight phase.

**Figure 24c:**
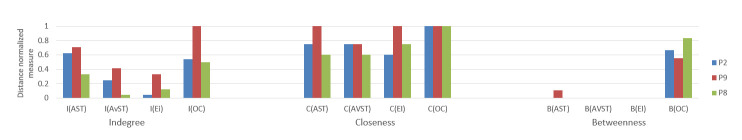
Distance normalized measure values for AOIs during Straight and Level flight phase.

**Figure 24d:**
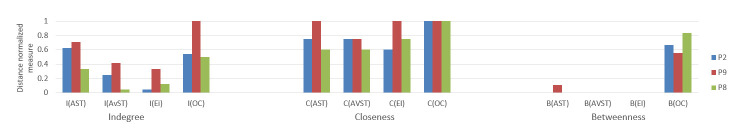
Distance normalized measure values for AOIs during Turn flight phase.

**Figure 24e:**
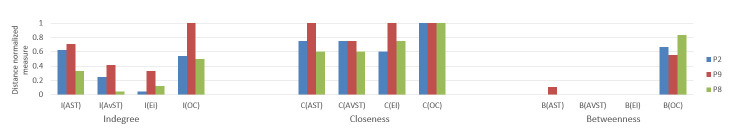
Distance normalized measure values for AOIs during five flight phases, I(“AOI”), C(“AOI”) and B(“AOI”) refers to normalized indegree, closeness and betweenness values, respectively. Different participants are represented with different colors.

Figures 24 from a) through e) shows the bar plot representations of the relative importance measures of four AOIs during five flight phases for each of three participants.

Examining relative importance measures of AOIs for each participant across five flight phases in Figure 24 a) to e) shows some trends for each participant, thus suggesting the presence of saccade strategies. High-performance participant P2 had high indegree and closeness values for AST and OC AOIs, on the other hand, his betweenness values for AST AOI were insignificant, except in the case of landing (Figure 24 e)). His betweenness values for OC AOI were constant. We should take into account that participant P2, according to Table 6, had the highest number of fixations. Since Figure 21 (P2) revealed that the EF durations were not high, the duration of gazes was most probably very short. If we take into account that only this participant gave advance to AOI AST, we can conclude that he had specific saccade strategy.

Participant P9 was trying to spread his attention evenly across all AOIs, which is evident from Figure 22 (P9). Figure 24 a) to e) shows that he had the highest levels of indegree and closeness for all AOIs across all flight phases (except for indegree during the turn). His centrality values are almost identical for observed AOI across all flight phases, apart from being high. He also managed to reach some values of betweenness for AST AOI. His saccade strategy is also distinguishable.

Low-performance participant P8 had different values of each importance measure for every AOI across five flight phases Figure 24 a) to e). Dominant AOI in his saccade strategy was OC, based on indegree and betweenness values. In some cases, indegree value for his OC AOI was highest among all participants Figure 24 b), c), e)), on the other hand during Take-off and Turn indegree values were lowest. The fact that he had the lowest number of fixations (Table 6), and that the EF durations were low (Figure 23), makes us conclude that he did not place his attention to desired AOIs and that he had an absence of saccade strategy.

## 6 Discussion

Results of the first part of this research showed that there is a positive correlation between the number of Fixation and Revisits count and performance during Psychological selection, which is the first stage of the overall selection process for cadet pilots at the Military Academy.

The conclusion is that the participants rated as High-performance during psychological selection did achieve some better results in the eye-tracking experiment because they made more fixations inside AST AOI, but that is not confirmation that they had better scanning technique.

In the reference literature, it is said by Katoh^(41)^ that the pilots exhibited the shortest dwell times during landing. This finding suggests that visual scanning patterns during landing are distinctly different from other periods of flight. Regarding fixations count, bars inside the Charts that represent number of fixations during landing phase inside AOI AST (Figure 13) and inside AOI OC (Figure 15) are indicating the same. Katoh^(41)^ also suggested that landings should require the attention of both instruments and the outside view. Hence, pilots must have more active visual scan patterns. The confirmation of an active scan patterns hypothesis might be the number of revisits given in Figure 17 and Figure 18.

We can see that, except for climb and level flight phases, in all other phases High-performance participants had a most significant number of revisits inside OC AOI. (Figure 18) Average-performance participants had less, and Low-performance participants had the least. Figure 18 shows that Specifically, the Take-off and Landing phase bars show that the Low-performance participants had a low number of revisits inside OC AOI. This is the pilot aptitude which is not desirable according to Kasarkis, Stehwien, Hickox, & Aretz^(31)^.

The analysis of the EM metrics using only the number of fixations, (which was the standard approach in many of the studies shown in Table 1) and introduced (measure) count of revisits failed to prove correlation with flight screening results. The correlation with psychological selection results exists only the former is not ample for selection of future pilots. The environments of the classroom (where the psychological tests are done) and the cockpit are not the same. The live training is where the personality pattern (confidence, making judgement under stress) comes in focus. The complete psychological selection rank depends on Personality inventory and Psychomotor testing, as well. The categorization given in this research follows only the pattern of Cognitive tests set data, described earlier. Psychomotor skills developed during flight screening process, and displayed during final flight with examiner, are the prevailing ones, when deciding whether the candidate would be given chance to become a pilot or not. The rank of the candidates is estimated including all other grades (flight screening and psychological selection).

The new DWN approach (procedure from reference literature) applied in event-based time intervals enabled us to visualize saccade strategies. Network visualization of one AOI sequence for each of the chosen participants showed how EM flows between AOIs. Despite relatively small EF numbers and EF durations, we could tell with certainty that the difference between participants existed, due to a vertex representation model. Besides, the bar plot representation of normalized importance measures for all AOIs and three participants across all flight phases enabled us to identify specific saccade strategy each of them had.

## 7. Conclusion

This study indicates that there is some potential in the correlation of psychological tests and the number of fixations and revisits, as predictors. Considering this, a part of the primary objective of this work that was to determine the correlation of eye-tracking measurements in a flight-simulated environment with the standard set of psychological test results was fulfilled. However, these predictors (revisits, fixations) do not seem to correlate well statistically with the Live flight performance. It should be pointed out that this could be due to a small sample available.

To overcome the shortcomings of traditional approach and to certify the difference between the test subjects we implemented additional network representation method with three target importance measures (indegree, closeness and betweenness). Implementation of this approach included plotting the graph objects with vertices of different colors and sizes, and with weighted and directed edges. Comparing the aforementioned network visualizations and normalized importance measures in bar plot graphs with the addition of a previously detected number of fixations confirmed the difference in saccade strategies between subjects. This could suggest the implementation of the eye-tracking device in the pilot selection process with the application of more refined EM metric visualization instead of the traditional one.

## 8 Limitations and future work

The first challenge we encountered was the dilemma between the use of event-based time intervals (flight phases) or evaluation of EM metrics over the whole time of the recorded flight. Using the whole period of recorded flight would certainly generate a greater sample of EF metrics numbers, namely fixations and revisits. Most of the time spent in 15-minute sortie is in straight-level-flight, especially for beginner pilot who adjusts the flight elements for an upcoming event (flight phase). Unintended TFs we mentioned earlier would certainly be a great deal of that time. Generated quantity could lead us to wrong conclusions about the quality of the saccade strategies for some participants, even though we introduced the measure of revisits to eliminate it. Shorter duration of event-based intervals, on the other hand, is connected with smaller EF numbers, and possibly invalid sample. Choosing event-based intervals over the whole flight demanded more recorded trials to solve the problem. Additionally, the choice of event-based intervals complies with demands during flights with the examiner. In our opinion, future work would request the investigation inside preset scenarios and participants performing only one flight phase with multiple trials. This would keep their focus only on flight elements, and not on flight organization itself.

Furthermore, the problem of selecting the number of different AOIs was also, the problem of their size and not only the importance. Electronic flight instruments layout represents the integration of several instruments that had a different shape and position earlier. With this integration, they had lost their position and shape, and another logic is used to draw attention. PFD provides most of the data necessary for flight. The multifunctional display MFD is not crucial for VFR flights. Creating more AOIs inside PFD would simply lead to their minimization and an additional decrease in EF numbers. Besides, the more precise eye-movement device would be required. Future work in this field could be the comparison of EM metrics when flying the traditional instrument layout with a digital one. Flight scenario would certainly have to be in Instrument Flight Rules (IFR).

## 9 Ethics and Conflict of Interest

The author(s) declare(s) that the contents of the article are in agreement with the ethics described in http://biblio.unibe.ch/portale/elibrary/BOP/jemr/ethics.html and that there is no conflict of interest regarding the publication of this paper.
